# Operations on Uterine Fibroids

**Published:** 1901-10-12

**Authors:** 


### OPERATIONS ON UTERINE FIBROIDS.

Speaking at Cheltenham on the subject of uterine fibroids and their treatment by operation, Dr. William Duncan said that the conditions calling for operation are four, namely (1) when the tumours cause severe hæmorrhage; (2) when they cause pressure symptoms; (3) when they are growing rapidly; and (4) when they complicate pregnancy. The operation which Dr. Duncan advises where operative treatment is required is that of intraperitoneal hysterectomy, meaning thereby an operation in which most of the uterus is removed, leaving a stump, consisting of a portion of the cervix, which is carefully shut off from the peritoneal cavity by means of peritoneal flaps sewn together after the method of Lembert. Of course in some cases in which the fibroid involves the whole cervix it is necessary to perform panhysterectomy, but except in those rare cases he fails to see what advantage is gained by removing the whole of the cervix and thus making a free communication between the vagina and the peritoneum. Dr. Duncan is quite opposed to vaginal hysterectomy for fibroids because he is confident that a fibroid which can be got out *per vaginam* can be removed by intraperitoneal hysterectomy quite as safely, and there is the great advantage in the abdominal operation of being able to see whether there be adhesions of the bowel or omentum to the tumour, or whether the tubes contain pus. He has performed intraperitoneal hysterectomy 127 times with four deaths, and his last 81 consecutive cases have all been successful.

